# Osteoporosis-Associated Mortality in Postmenopausal Women in the United States From 1999 to 2023: A CDC WONDER-Based Study

**DOI:** 10.7759/cureus.87721

**Published:** 2025-07-11

**Authors:** Muhammad Shabir, Muhammad Yasin Khan, Muhammad Younas Khan, Murad Ali, Rahman Syed, Ameer Afzal Khan, Anfal Khan, Fazal Syed, Mohammad Idrees, Muhammad Tariq

**Affiliations:** 1 Orthopaedics, Saidu Group of Teaching Hospitals, Swat, PAK; 2 Internal Medicine, Swat Medical College, Swat, PAK; 3 Internal Medicine, Saidu Medical College, Swat, PAK; 4 Internal Medicine, Northwest School of Medicine, Peshawar, PAK

**Keywords:** cdc-wonder, mortality trends, osteoporosis, postmenopausal women, united states

## Abstract

Background and Aim: Osteoporosis remains a significant contributor to illness and death among postmenopausal women, primarily due to complications from fractures. This study examined national trends and disparities in osteoporosis-related mortality over a 24-year period. Methods: Mortality records of postmenopausal women from 1999 to 2023 were analyzed using the Centers for Disease Control and Prevention Wide-Ranging Online Data for Epidemiologic Research (CDC WONDER) database to assess osteoporosis-related deaths. Age-adjusted mortality rates (AAMRs) were calculated and examined across time, demographic groups, geographic regions, and fracture involvement. Statistical trend analysis was used to evaluate changes in mortality patterns over time. Results: A total of 232,877 osteoporosis-related deaths were recorded. The overall AAMR declined from 29.35 in 1999 to 12.00 in 2023 (average annual percent change (AAPC): -3.75%; 95% CI: -4.71 to -2.77; p < 0.000001). Mortality due to osteoporosis with pathological fracture showed a sharper decline (AAPC: -5.14%) compared to osteoporosis without fracture (AAPC: -3.62%). White women had the highest AAMRs throughout, though all racial/ethnic groups experienced significant reductions. Regional analysis revealed the highest mortality rates in the Midwest and West, with Vermont reporting the highest state-level AAMR (74.97). Recent years (2018-2021) showed non-significant increases in mortality across subgroups, which may be associated with healthcare disruptions during COVID-19. Conclusion: Osteoporosis-related mortality among postmenopausal women significantly declined over the past 25 years, reflecting advances in diagnosis, treatment, and prevention. However, persistent racial, geographic, and fracture-related disparities underscore the need for targeted public health interventions and equitable access to osteoporosis care.

## Introduction

Osteoporosis is a progressive systemic skeletal disease characterized by low bone mass and microarchitectural deterioration of bone tissue, leading to increased bone fragility and susceptibility to fractures. Although both men and women are affected, postmenopausal women are at particularly high risk due to the sharp decline in estrogen levels, which accelerates bone resorption. As women transition into menopause, they face a physiological shift that greatly increases the likelihood of skeletal complications. In the United States (US), it is estimated that between one-third and one-half of women over the age of 50 will experience an osteoporotic fracture in their lifetime, including fractures of the hip, spine, or wrist [1].

While osteoporosis is often said to be a silent, degenerative disease, the clinical consequences of its complications can be both catastrophic and enduring. Hip and vertebral fractures are among the most severe, not only because of the high associated mortality (up to 24% of women over 50 die within one year of a hip fracture) but also because of the cascade of other serious outcomes that follow [2]. Fractures often result in chronic pain, kyphosis, and spinal impairment, which impair mobility and posture and contribute to long-term disabilities [3]. Vertebral compression fractures can lead to a decrease in height, stooped posture (Dowager's hump), and altered biomechanics, which further increase the risk of falls and subsequent fractures [4].

Beyond physical symptoms, osteoporosis and its complications severely impact mental health and quality of life. Many patients develop depression, anxiety, and fear of falling, which leads to social withdrawal and isolation. These psychosocial effects are compounded by the loss of independence, especially among older adults who may become reliant on caregivers or require institutionalization after a major fracture [5]. Complications such as immobility-related thromboembolic events, pressure ulcers, and hospital-acquired infections (e.g., pneumonia, urinary tract infections (UTIs), and sepsis) further contribute to morbidity and mortality, particularly following hip fractures. Studies report that one-year mortality rates after hip fracture can reach 24%, and up to 30% of patients experience at least one serious postoperative complication [6,7].

Over the past two decades, initiatives in the US healthcare system, including expanded bone mineral density (BMD) testing and fracture prevention strategies, have aimed to reduce the burden of osteoporosis. However, disparities in access, awareness, and adherence to treatment persist. The aging US population is projected to include over 61 million women aged 50 and older by 2030, intensifying the need for improved prevention, diagnosis, and management of osteoporosis [8].

Despite the availability of effective pharmacological therapies, osteoporosis remains underdiagnosed and undertreated, especially after the first fracture [9]. Women from racial and ethnic minority groups, as well as those in rural or socioeconomically disadvantaged areas, face a compounded risk due to healthcare inequities. The presence of comorbidities, such as diabetes, cardiovascular disease, and cognitive impairment, further complicates recovery and increases mortality risk [10].

Our study uses national data from the Centers for Disease Control and Prevention Wide-Ranging Online Data for Epidemiologic Research (CDC WONDER) database to evaluate osteoporosis-related mortality in postmenopausal women from 1999 to 2023. By examining temporal trends, racial and geographic disparities, and systemic implications, this study aims to inform future interventions that better address the full burden of osteoporosis in the US.

## Materials and methods

Study setting and population

This descriptive study used the CDC WONDER database death certificate data from 1999 to 2023, to examine osteoporosis-related mortality in postmenopausal women. For the purpose of this study, postmenopausal women were defined as females aged 55 years and older, consistent with epidemiologic norms. Data were analyzed using codes from the International Statistical Classification of Diseases and Related Health Problems, 10th Revision (ICD-10), specifically codes M80 (osteoporosis with pathological fracture) and M81 (osteoporosis without pathological fracture). Previous research, particularly on mortality rates from cardiovascular disorders, supported the approach utilized in this study, proving its trustworthiness [11]. The dataset contains death certificate data from all 50 states and the District of Columbia, including both underlying and contributing causes of death. For the analysis, deaths involving osteoporosis in postmenopausal women were recognized if they appeared anywhere on the death certificate, whether as a contributory or underlying cause. This study was exempt from institutional review board approval since it made use of publicly available, de-identified government data. The study follows the Strengthening the Reporting of Observational Studies in Epidemiology (STROBE) guidelines for reporting observational research.

Data extraction

Data on population size, year, place of death, demographics, census region, and state were gathered for analysis. Race/ethnicity categories included non-Hispanic (NH) White, Hispanic or Latino, NH American Indian or Alaskan Native, and NH Asian or Pacific Islander. These demographic categories were generated from information recorded on death certificates, which has been used in previous studies using the WONDER database [12]. The country was divided into four areas based on the definitions provided by the US Census Bureau: Northeast, Midwest, South, and West.

Statistical analysis

To analyze national trends in osteoporosis-related mortality in postmenopausal women, we calculated age-adjusted mortality rates (AAMRs) per 100,000 population from 1999 to 2023, stratified by year, race/ethnicity, state, and census region, with 95% CIs. AAMRs were calculated by adjusting the osteoporosis-related deaths in postmenopausal women to the 1999 US population standard. In order to evaluate the annual trends in mortality, we used the Joinpoint Regression Program (Joinpoint version 4.9.0.0, National Cancer Institute) to estimate the annual percent change (APC) in AAMR, with 95% CI [13]. This approach uses log-linear regression models to detect shifts in trends, and APCs were considered to be increasing or decreasing if the slope indicating the change in mortality was significantly different from zero, as determined by two-tailed t-tests; a p-value of < 0.05 was considered to be statistically significant.

## Results

**Figure 1:**
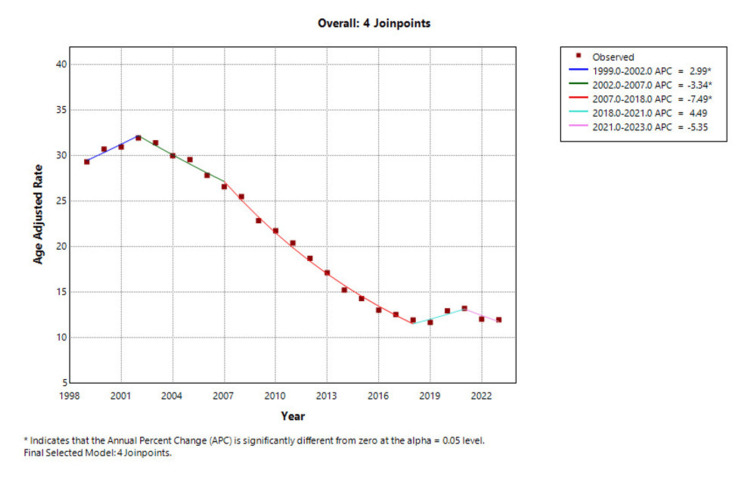
Overall osteoporosis-related mortality in postmenopausal women, AAMRs per 100,000 in the US, 1999-2023 AAMR: Age-adjusted mortality rate

From 1999 to 2023, a total of 232,877 deaths were attributed to osteoporosis among postmenopausal women in the US. During this period, AAMR declined substantially, decreasing from 29.35 per 100,000 in 1999 to 12.00 per 100,000 in 2023. Between 1999 and 2002, mortality rates increased significantly, with an APC of 2.99% (95% CI: 0.40 to 5.63; p = 0.0269). This was followed by a statistically significant decline from 2002 to 2007 (APC: -3.34%; 95% CI: -4.85 to -1.80; p = 0.0006). The most pronounced reduction occurred between 2007 and 2018, with an APC of -7.49% (95% CI: -7.92 to -7.06; p < 0.0001). A non-significant increase was observed from 2018 to 2021 (APC: 4.49%; 95% CI: -2.33 to 11.79; p = 0.1795), followed by a non-significant decline from 2021 to 2023 (APC: -5.35%; 95% CI: -11.59 to 1.32; p = 0.1033), as shown in Figure 1 and Appendix A. Overall, the average APC (AAPC) for the entire study period was -3.75% (95% CI: -4.71 to -2.77; p < 0.000001), indicating a significant long-term decline in osteoporosis-related mortality among postmenopausal women in the US.

**Figure 2:**
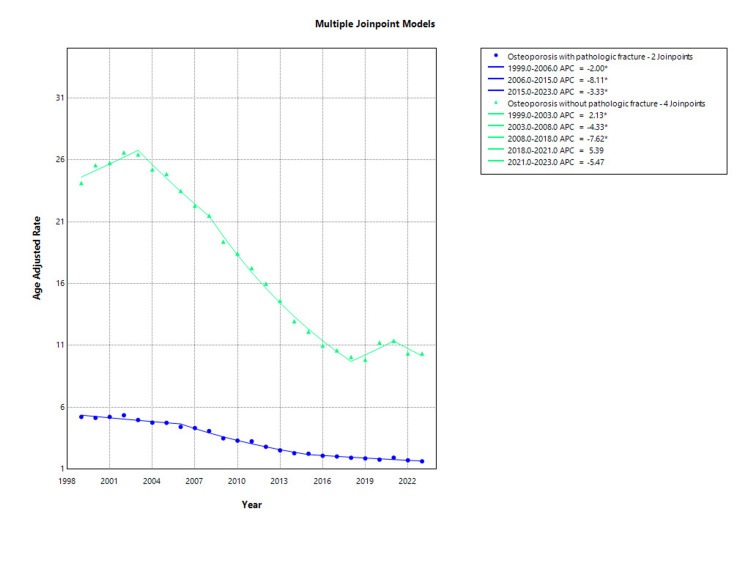
Osteoporosis with pathological fracture and without fracture stratified AAMR per 100,000 in the US, 1999-2023 AAMR: Age-adjusted mortality rate

From 1999 to 2023, a total of 36,933 deaths were attributed to osteoporosis with pathological fractures. Over this period, AAMR decreased substantially from 5.22 per 100,000 in 1999 to 1.63 per 100,000 in 2023. Between 1999 and 2003, mortality remained relatively stable for osteoporosis with pathological fractures, with a non-significant APC of -0.18% (95% CI: -5.05 to 4.93; p = 0.939). However, from 2003 to 2023, there was a statistically significant and sustained decline in mortality, with an APC of -6.10% (95% CI: -6.61 to -5.59; p < 0.001) as shown in Figure 2. The overall AAPC from 1999 to 2023 was -5.14 % (95% CI: -5.98 to -4.29; p < 0.001).

During the study period, a total of 196,102 deaths were attributed to osteoporosis without pathological fracture. Over this period, the AAMR decreased from 24.13 per 100,000 in 1999 to 10.34 per 100,000 in 2023. From 1999 to 2003, mortality rates increased modestly but significantly, with an APC of 2.13% (95% CI: 0.37 to 3.91; p = 0.022). This was followed by a significant decline between 2003 and 2008 (APC: -4.33%; 95% CI: -5.98 to -2.66; p < 0.001) and an even steeper decline from 2008 to 2018 (APC: -7.62%; 95% CI: -8.18 to -7.06; p < 0.001). A non-significant increase in mortality occurred from 2018 to 2021 (APC: 5.39%; 95% CI: -2.00 to 13.33; p = 0.141), followed by a non-significant decline from 2021 to 2023 (APC: -5.47%; 95% CI: -12.13 to 1.69; p = 0.118) as shown in Figure 2 and Appendix B. Overall, the AAPC from 1999 to 2023 was -3.62% (95% CI: -4.66 to -2.57; p < 0.001).

**Figure 3:**
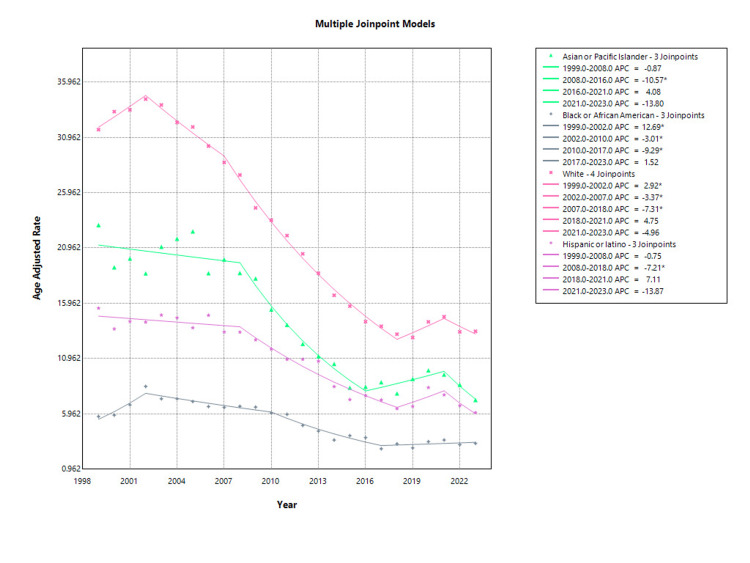
Race-stratified osteoporosis-related mortality in postmenopausal women, AAMRs per 100,000 in the US, 1999-2023 AAMR: Age-adjusted mortality rate

Among Asian or Pacific Islander postmenopausal women, AAMR due to osteoporosis declined from 23.03 per 100,000 in 1999 to 7.21 in 2023. The trend from 1999 to 2008 was not statistically significant (APC: -0.87%; 95% CI: -2.89 to 1.20; p = 0.381). However, a significant decline occurred between 2008 and 2016 (APC: -10.57%; 95% CI: -12.99 to -8.09; p < 0.001). This was followed by a non-significant increase from 2016 to 2021 (APC: 4.08%; 95% CI: -2.03 to 10.57; p = 0.178) and another non-significant decline from 2021 to 2023 (APC: -13.80%; 95% CI: -29.03 to 4.70; p = 0.124) as shown in Figure 3.

In Black or African American postmenopausal women, the AAMR increased from 5.72 in 1999 to a peak of 8.44 in 2002, followed by a decline to 3.29 by 2023. From 1999 to 2002, there was a significant rise in mortality (APC: 12.69%; 95% CI: 1.68 to 24.90; p = 0.026). This was followed by a significant decrease from 2002 to 2010 (APC: -3.01%; 95% CI: -5.39 to -0.57; p = 0.019) and an even steeper decline from 2010 to 2017 (APC: -9.29%; 95% CI: -12.37 to -6.10; p < 0.001). The trend from 2017 to 2023 was not statistically significant (APC: 1.52%; 95% CI: -2.25 to 5.43; p = 0.407) as shown in Figure 3.

White postmenopausal women had the highest AAMRs across all racial groups, starting at 31.66 in 1999 and decreasing to 13.42 in 2023. From 1999 to 2002, mortality rose significantly (APC: 2.92%; 95% CI: 0.21 to 5.69; p = 0.037). A significant decline followed from 2002 to 2007 (APC: -3.37%; 95% CI: -4.96 to -1.76; p = 0.001) and continued steeply from 2007 to 2018 (APC: -7.31%; 95% CI: -7.77 to -6.85; p < 0.001). Trends from 2018 to 2021 (APC: 4.75%; p = 0.184) and 2021 to 2023 (APC: -4.96%; p = 0.151) were not statistically significant as shown in Figure 3.

Among Hispanic or Latino postmenopausal women, the AAMR declined from 15.52 in 1999 to 6.07 in 2023. From 1999 to 2008, the trend was not statistically significant (APC: -0.75%; 95% CI: -2.51 to 1.05; p = 0.385), but a substantial and significant decrease was observed from 2008 to 2018 (APC: -7.21%; 95% CI: -8.73 to -5.66; p < 0.001). The subsequent trends from 2018 to 2021 (APC: 7.11%; p = 0.407) and 2021 to 2023 (APC: -13.87%; p = 0.094) were not statistically significant, indicating a possible plateau in recent years as shown in Figure 3.

**Figure 4:**
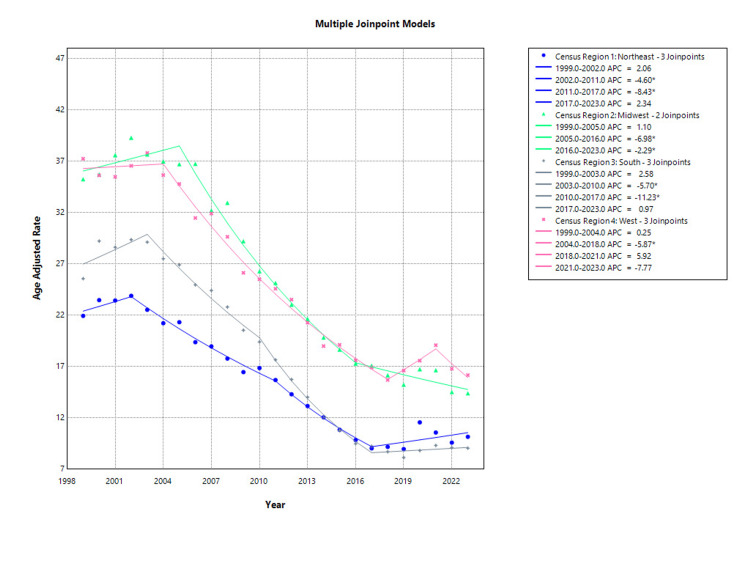
Region-stratified osteoporosis-related mortality in postmenopausal women, AAMRs per 100,000 in the US, 1999-2023 AAMR: Age-adjusted mortality rate

In the Northeast, the AAMR increased slightly from 21.94 in 1999 to 23.89 in 2002, followed by a significant decline to 15.68 in 2011 (APC: -4.60%; 95% CI: -6.07 to -3.10; p < 0.001) and a steeper drop to 9.02 in 2017 (APC: -8.43%; 95% CI: -11.94 to -4.77; p < 0.001). Between 2017 and 2023, mortality saw a non-significant increase to 10.15 (APC: 2.34%; 95% CI: -0.87 to 5.65; p = 0.142), while the earlier increase from 1999 to 2002 was also non-significant (APC: 2.06%; p = 0.532) as shown in Figure 4.

Postmenopausal women in the Midwest experienced high initial mortality with an AAMR of 35.26 in 1999, which slightly increased to 36.72 by 2005. This early rise was not statistically significant (APC: 1.10%; 95% CI: -0.58 to 2.80; p = 0.186). A significant and consistent decline followed from 2005 to 2016 (APC: -6.98%; 95% CI: -7.79 to -6.17; p < 0.001), with the AAMR reaching 17.29. A further significant reduction occurred between 2016 and 2023 (APC: -2.29%; 95% CI: -4.07 to -0.48; p = 0.016), bringing the AAMR down to 14.39 in 2023 as shown in Figure 4.

In the South, the AAMR rose from 25.57 in 1999 to a peak of 29.13 in 2003, although this increase did not reach statistical significance (APC: 2.58%; 95% CI: -0.10 to 5.34; p = 0.058). A significant decrease followed from 2003 to 2010 (APC: -5.70%; 95% CI: -7.06 to -4.32; p < 0.001), and an even sharper drop occurred between 2010 and 2017 (APC: -11.23%; 95% CI: -12.84 to -9.59; p < 0.001), reaching an AAMR of 9.22. From 2017 to 2023, the rate remained stable with a non-significant change (APC: 0.97%; 95% CI: -1.11 to 3.08; p = 0.337), ending at 9.04 as shown in Figure 4.

In the Western region, AAMR for osteoporosis-related deaths among postmenopausal women showed initial stability, with a non-significant change from 37.25 in 1999 to 35.66 in 2004 (APC: 0.25%; 95% CI: -1.66 to 2.19; p = 0.787). A significant and consistent decline was then observed from 2004 to 2018, during which the AAMR dropped to 15.69 (APC: -5.87%; 95% CI: -6.34 to -5.40; p < 0.001). This trend temporarily reversed from 2018 to 2021 with a non-significant rise to 19.08 (APC: 5.92%; 95% CI: -4.32 to 17.26; p = 0.245), followed by a subsequent non-significant decline to 16.16 in 2023 (APC: -7.77%; 95% CI: -16.74 to 2.16; p = 0.112) as shown in Figure 4. 

**Figure 5:**
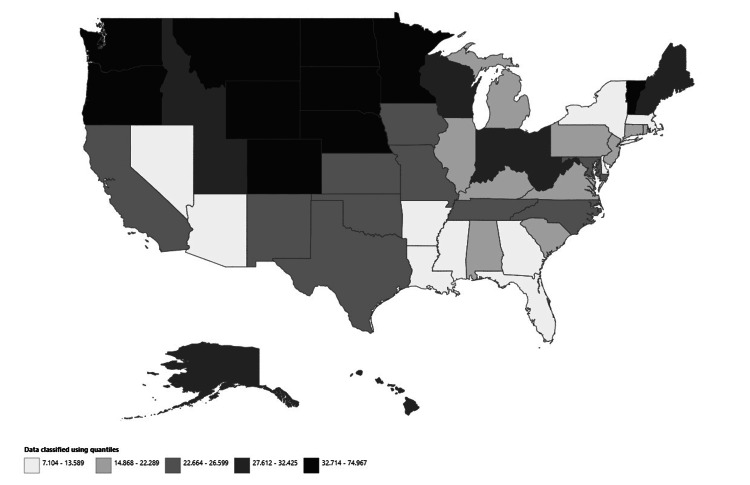
State-stratified osteoporosis-related mortality in postmenopausal women, AAMRs per 100,000 in the US, 1999-2023 AAMR: Age-adjusted mortality rate

Among US states, Vermont reported the highest AAMR due to osteoporosis in postmenopausal women, with a rate of 74.97 per 100,000 population. This was followed by Nebraska (46.38), Montana (45.73), Minnesota (45.42), and Wyoming (43.80) as shown in Figure 5 and Appendix C.

## Discussion

Overall mortality trends and public health impact

The findings of this comprehensive 25-year analysis reveal a substantial decline in osteoporosis-related mortality among postmenopausal women in the US, with AAMR decreasing from 29.35 per 100,000 in 1999 to 12.00 per 100,000 in 2023, representing an overall 59% reduction. This significant improvement aligns with global trends in osteoporosis management and reflects the overall impact of enhanced clinical awareness, improved diagnostic capabilities, and the widespread adoption of evidence-based therapeutic interventions [14].

The observed AAPC of -3.75% over the study period demonstrates sustained progress in reducing osteoporosis-related mortality, which is particularly noteworthy given that osteoporosis affects approximately 54 million adults aged 50 years and older in the US [15]. This mortality reduction has significant public health implications, as osteoporosis-related complications, particularly hip fractures, are associated with excess mortality rates of 10-20% above age-expected levels (International Osteoporosis Foundation, 2025) [16].

Temporal patterns and healthcare evolution

The joinpoint regression analysis revealed distinct phases in mortality trends that likely reflect key developments in osteoporosis care and prevention strategies. The initial increase in mortality from 1999 to 2002 (APC: 2.99%) may reflect improved diagnostic coding practices and increased recognition of osteoporosis as a primary cause of death, rather than a true increase in disease-related mortality, also potentially because of improved reporting practices and increased recognition of osteoporosis-related deaths. This phenomenon has been observed in other chronic disease mortality studies where enhanced diagnostic awareness initially increases reported mortality rates [11].

The significant decline beginning in 2002 corresponds with several pivotal developments in osteoporosis management. The Women's Health Initiative findings published in 2002, while controversial for hormone replacement therapy, heightened awareness of postmenopausal bone health issues [17]. Additionally, the early 2000s saw the introduction and widespread adoption of bisphosphonate therapy, which has been shown to reduce fracture risk by 40-70% in clinical trials [18].

The most pronounced mortality reduction occurred between 2007 and 2018 (APC: -7.49%), coinciding with the maturation of osteoporosis screening guidelines, fracture liaison services, and evidence-based treatment protocols [19]. This period also saw the approval and introduction of newer pharmacologic agents, including teriparatide, denosumab, and zoledronic acid, expanding treatment options for high-risk patients [20]. While this temporal alignment is suggestive, we recognize that the population-level impact of new therapies likely involves a lag due to adoption rates, prescribing behaviors, and patient adherence. Thus, the mortality decline likely reflects a cumulative and gradual effect of multiple interventions rather than an immediate therapeutic response.

Fracture-specific mortality patterns

The analysis of osteoporosis mortality by fracture status reveals important clinical insights. Deaths attributed to osteoporosis with pathological fractures showed a more consistent decline pattern (AAPC: 5.14%) compared to those without documented fractures (AAPC: -3.62%). This differential suggests that improvements in acute fracture care, including enhanced surgical techniques, multidisciplinary care teams, and post-fracture rehabilitation programs, have been particularly effective in reducing mortality among patients with established fractures.

The sustained decline in fracture-related mortality from 2003 (APC: -6.10%) likely reflects the implementation of systematic approaches to fracture prevention, including the development of fracture liaison services and secondary fracture prevention programs. However, from 2018 to 2021, there was a non-significant increase in mortality (APC: 4.49%), likely influenced by healthcare access disruptions during COVID-19, which led to decreased routine care, including osteoporosis screening and treatment initiation [21]. These initiatives have been shown to reduce subsequent fracture risk by up to 50% and improve overall survival outcomes [22]. While the distinction between osteoporosis-related deaths with and without pathological fracture provides useful clinical stratification, it is important to acknowledge that this categorization may be influenced by variability in diagnostic coding practices. The absence of a documented fracture does not conclusively rule out a fracture event, as it may have been omitted from the death certificate or not coded accurately. Therefore, differences in mortality trends between M80 and M81 cases may partly reflect administrative or documentation inconsistencies rather than distinct biological or clinical pathways.

Racial and ethnic disparities in mortality outcomes

The racial and ethnic disparities observed in this study highlight persistent inequities in osteoporosis care and outcomes. White postmenopausal women demonstrated the highest AAMRs throughout the study period, which is consistent with established epidemiological patterns showing higher osteoporosis prevalence and fracture rates in Caucasian populations [23].

However, the mortality patterns among racial and ethnic minorities reveal concerning disparities in care quality and outcomes. Despite having lower baseline osteoporosis rates, Black and African American women showed variable mortality trends, including a significant initial increase from 1999 to 2002 (APC: 12.69%). This pattern may reflect delayed diagnosis, reduced access to preventive care, and disparities in treatment quality that have been well-documented in the literature [24,25].

The observation that Black women experience poorer outcomes following hip fractures, including increased morbidity and mortality compared to their White counterparts, underscores the need for targeted interventions to address these disparities [26]. Potential contributors to these disparities may include socioeconomic barriers, limited access to specialized care, delayed diagnosis due to lower screening rates, and possible biological differences in bone metabolism and fracture healing [27]. While these factors have been proposed in prior studies, further research is needed to establish their specific roles in mortality outcomes.

Asian or Pacific Islander women demonstrated a particularly steep decline in mortality between 2008 and 2016 (APC: 10.57%), which may reflect improved access to care, cultural adaptation of osteoporosis awareness programs, and potentially genetic factors affecting treatment response. However, the subsequent non-significant trends suggest that continued efforts are needed to maintain these improvements.

Hispanic or Latino women showed substantial mortality reductions from 2008 to 2018 (APC: 7.21%), but the recent plateau in mortality trends (2018-2023) warrants close monitoring and may indicate emerging challenges in maintaining care continuity or access to newer therapeutic options within this population.

Geographic variations and healthcare access

The regional analysis reveals significant geographic disparities in osteoporosis mortality that likely reflect differences in healthcare infrastructure, specialist availability, and population demographics. The consistently high mortality rates in the Midwest (AAMR: 36.72) and (AAMR: 37.25) West regions may be related to rural-urban healthcare disparities, with rural populations having limited access to endocrinologists, rheumatologists, and specialized osteoporosis centers.

The West showed a significant drop between 2004 and 2018 (APC: -5.87%; p < 0.001), while the Midwest had a steeper decline from 2005 to 2016 (APC: -6.98%; p < 0.001), reflecting possible regional differences in public health interventions, clinical practice, and access to specialized bone health services. Conversely, the Southern region had persistently high mortality and saw a peak AAMR of 29.13 in 2003. Although a significant decline followed through 2017, recent trends suggest stabilization, potentially due to slower adoption of preventive strategies and greater healthcare inequities [28]. The Northeast, while initially lower in mortality, showed modest improvements and has remained relatively stable in recent years

Vermont's notably high AAMR of 74.97 per 100,000 is striking and may be influenced by a combination of factors, such as an aging population, rural healthcare infrastructure, or limited access to osteoporosis screening and treatment services. The clustering of high-mortality states in the northern Midwest (Nebraska, Montana, Minnesota, Wyoming) suggests regional factors such as climate-related vitamin D deficiency, rural healthcare access issues, or population-specific risk factors that warrant further investigation.

The significant mortality reductions observed in all regions demonstrate that improvements in osteoporosis care have been widespread, but the persistent regional variations indicate that targeted interventions may be needed to address geographic disparities in care access and outcomes.

Clinical implications

The overall decline in osteoporosis mortality observed in this study represents a significant public health achievement, but several important clinical implications emerge from these findings. The recent non-significant increases in mortality from 2018 to 2021 across multiple demographic groups warrant careful attention and may reflect several factors, including healthcare disruptions during COVID-19, changes in care-seeking behavior, or the emergence of treatment-resistant patient populations.

The pandemic likely had multiple impacts on osteoporosis care, including delayed routine screening, postponed elective procedures, reduced physical activity leading to accelerated bone loss, and potential medication adherence issues due to healthcare access limitations [29]. These factors may have contributed to the observed temporary increase in mortality during this period.

Limitations and future research needs

Several limitations should be considered when interpreting these findings. First, the analysis relies on death certificate data, which may be subject to misclassification, underreporting, or overreporting of osteoporosis, particularly when other competing conditions are listed as the primary cause of death. Our definition of osteoporosis-related mortality includes both underlying and contributory causes of death, which may overestimate the direct causal role of osteoporosis. Second, the data are not linked to clinical or hospitalization records, meaning we could not verify actual fracture events, treatment histories, or in-hospital complications. Third, we were unable to adjust for important individual-level confounders, including comorbidities (e.g., cardiovascular disease, diabetes) and socioeconomic status, which are known to influence both fracture risk and mortality outcomes.

Future research should aim to elucidate the underlying factors contributing to the recent plateau in osteoporosis-related mortality, particularly during and after COVID-19. This includes examining disruptions in preventive care, delays in fracture management, and medication non-adherence during public health emergencies. Additionally, long-term studies are needed to evaluate the sustained impact of the pandemic on osteoporosis screening, treatment initiation, and post-fracture outcomes. Future investigations should also explore the persistent racial, ethnic, and geographic disparities identified in this study to guide the development of equitable, population-specific interventions. Lastly, future work could benefit from expressing osteoporosis-related deaths as a proportion of all-cause mortality among postmenopausal women to better contextualize its relative burden within the aging population. 

## Conclusions

This comprehensive analysis demonstrates substantial progress in reducing osteoporosis-related mortality among postmenopausal women in the US over the past 25 years. The observed reduction in AAMR reflects the collective impact of improved diagnostic capabilities, evidence-based treatment protocols, and enhanced clinical awareness. However, persistent disparities across racial, ethnic, and geographic lines highlight the need for continued efforts to ensure equitable access to high-quality osteoporosis care for all populations. The recent trends suggesting mortality plateaus in some groups underscore the importance of ongoing surveillance and the development of innovative approaches to continue improving outcomes for women at risk of osteoporosis-related complications. Future efforts may benefit from national-level monitoring systems that integrate electronic health record data, Medicare claims, or fracture registries to detect emerging disparities. Policy interventions aimed at improving access to screening and post-fracture care, particularly in underserved populations, will also be critical to sustaining progress.

## Appendices

Appendix A 

**Table 1:** Overall osteoporosis-related deaths, crude rate, and AAMR among postmenopausal women in the US from 1999 to 2023 AAMR: Age-adjusted mortality rate

Overall	Year	Deaths	Crude Rate	AAMR	AAMR Lower 95% CI	AAMR Upper 95% CI	AAMR Standard Error
Overall	1999	11,142	33.891	29.353	28.806	29.9	0.279
Overall	2000	11,834	35.632	30.743	30.187	31.299	0.284
Overall	2001	12,083	35.825	30.987	30.432	31.542	0.283
Overall	2002	12,603	36.412	31.962	31.401	32.522	0.286
Overall	2003	12,550	35.443	31.434	30.881	31.986	0.282
Overall	2004	12,104	33.435	30.028	29.49	30.565	0.274
Overall	2005	12,188	32.878	29.59	29.062	30.119	0.27
Overall	2006	11,698	30.814	27.858	27.349	28.366	0.259
Overall	2007	11,423	29.376	26.61	26.118	27.102	0.251
Overall	2008	11,149	27.96	25.539	25.06	26.018	0.244
Overall	2009	10,193	24.905	22.888	22.439	23.338	0.229
Overall	2010	9,858	23.591	21.781	21.345	22.216	0.222
Overall	2011	9,544	22.116	20.435	20.018	20.851	0.213
Overall	2012	8,929	20.15	18.743	18.348	19.138	0.202
Overall	2013	8,315	18.289	17.155	16.78	17.53	0.191
Overall	2014	7,553	16.192	15.263	14.913	15.613	0.179
Overall	2015	7,204	15.061	14.335	13.999	14.672	0.172
Overall	2016	6,701	13.699	13.057	12.739	13.374	0.162
Overall	2017	6,582	13.156	12.578	12.27	12.886	0.157
Overall	2018	6,334	12.42	11.979	11.68	12.277	0.152
Overall	2019	6,275	12.083	11.7	11.408	11.992	0.149
Overall	2020	7,034	13.334	12.971	12.666	13.277	0.156
Overall	2021	6,681	12.732	13.254	12.935	13.573	0.163
Overall	2022	6,569	12.329	12.058	11.765	12.351	0.15
Overall	2023	6,331	11.764	12.005	11.709	12.302	0.151

Appendix B 

**Table 2:** Osteoporosis with pathological fracture and without fracture stratified deaths, crude rate, and AAMR in the US, 1999-2023 AAMR: Age-adjusted mortality rate

Osteoporosis With or Without Fracture	Year	Deaths	Crude Rate	AAMR	AAMR Lower 95% CI	AAMR Upper 95% CI	AAMR Standard Error
Osteoporosis with pathologic fracture	1999	1,985	6.038	5.222	4.991	5.453	0.118
Osteoporosis with pathologic fracture	2000	1,997	6.013	5.146	4.919	5.372	0.116
Osteoporosis with pathologic fracture	2001	2,053	6.087	5.223	4.997	5.45	0.116
Osteoporosis with pathologic fracture	2002	2,115	6.111	5.357	5.128	5.586	0.117
Osteoporosis with pathologic fracture	2003	2,005	5.662	4.974	4.756	5.193	0.112
Osteoporosis with pathologic fracture	2004	1,940	5.359	4.76	4.548	4.973	0.109
Osteoporosis with pathologic fracture	2005	1959	5.285	4.746	4.534	4.957	0.108
Osteoporosis with pathologic fracture	2006	1,862	4.905	4.415	4.213	4.617	0.103
Osteoporosis with pathologic fracture	2007	1,862	4.788	4.323	4.125	4.521	0.101
Osteoporosis with pathologic fracture	2008	1,786	4.479	4.08	3.889	4.271	0.098
Osteoporosis with pathologic fracture	2009	1,573	3.843	3.492	3.317	3.666	0.089
Osteoporosis with pathologic fracture	2010	1,503	3.597	3.304	3.134	3.473	0.086
Osteoporosis with pathologic fracture	2011	1,521	3.525	3.247	3.082	3.413	0.085
Osteoporosis with pathologic fracture	2012	1,345	3.035	2.809	2.656	2.962	0.078
Osteoporosis with pathologic fracture	2013	1,224	2.692	2.518	2.375	2.662	0.073
Osteoporosis with pathologic fracture	2014	1,154	2.474	2.289	2.154	2.423	0.069
Osteoporosis with pathologic fracture	2015	1,152	2.408	2.249	2.116	2.381	0.068
Osteoporosis with pathologic fracture	2016	1,081	2.21	2.083	1.957	2.209	0.064
Osteoporosis with pathologic fracture	2017	1,046	2.091	2.023	1.899	2.148	0.064
Osteoporosis with pathologic fracture	2018	1,030	2.02	1.918	1.799	2.036	0.06
Osteoporosis with pathologic fracture	2019	1,007	1.939	1.866	1.75	1.982	0.059
Osteoporosis with pathologic fracture	2020	961	1.822	1.766	1.653	1.879	0.057
Osteoporosis with pathologic fracture	2021	971	1.85	1.925	1.803	2.046	0.062
Osteoporosis with pathologic fracture	2022	938	1.761	1.715	1.605	1.825	0.056
Osteoporosis with pathologic fracture	2023	863	1.604	1.632	1.522	1.741	0.056
Osteoporosis without pathologic fracture	1999	9,157	27.853	24.131	23.634	24.627	0.253
Osteoporosis without pathologic fracture	2000	9,837	29.619	25.564	25.056	26.071	0.259
Osteoporosis without pathologic fracture	2001	10,030	29.738	25.764	25.257	26.27	0.258
Osteoporosis without pathologic fracture	2002	10,488	30.302	26.604	26.093	27.116	0.261
Osteoporosis without pathologic fracture	2003	10,545	29.781	26.436	25.929	26.943	0.259
Osteoporosis without pathologic fracture	2004	10,165	28.079	25.227	24.734	25.719	0.251
Osteoporosis without pathologic fracture	2005	10,229	27.594	24.852	24.367	25.336	0.247
Osteoporosis without pathologic fracture	2006	9,850	25.946	23.493	23.025	23.96	0.238
Osteoporosis without pathologic fracture	2007	9,570	24.611	22.284	21.834	22.735	0.23
Osteoporosis without pathologic fracture	2008	9,378	23.518	21.488	21.048	21.927	0.224
Osteoporosis without pathologic fracture	2009	8,638	21.106	19.395	18.981	19.809	0.211
Osteoporosis without pathologic fracture	2010	8,365	20.018	18.429	18.029	18.83	0.204
Osteoporosis without pathologic fracture	2011	8,039	18.628	17.25	16.867	17.633	0.195
Osteoporosis without pathologic fracture	2012	7,594	17.137	16.001	15.635	16.367	0.187
Osteoporosis without pathologic fracture	2013	7,100	15.617	14.606	14.261	14.952	0.176
Osteoporosis without pathologic fracture	2014	6,406	13.733	12.958	12.635	13.28	0.165
Osteoporosis without pathologic fracture	2015	6,059	12.667	12.115	11.805	12.425	0.158
Osteoporosis without pathologic fracture	2016	5,627	11.504	10.988	10.697	11.28	0.149
Osteoporosis without pathologic fracture	2017	5,537	11.067	10.599	10.316	10.882	0.144
Osteoporosis without pathologic fracture	2018	5,309	10.41	10.068	9.794	10.342	0.14
Osteoporosis without pathologic fracture	2019	5,272	10.151	9.841	9.573	10.109	0.137
Osteoporosis without pathologic fracture	2020	6,079	11.524	11.234	10.949	11.518	0.145
Osteoporosis without pathologic fracture	2021	5,715	10.891	11.377	11.081	11.674	0.151
Osteoporosis without pathologic fracture	2022	5,639	10.584	10.35	10.079	10.622	0.139
Osteoporosis without pathologic fracture	2023	5,474	10.171	10.348	10.073	10.622	0.14

Appendix C 

**Table 3:** State-stratified osteoporosis-related mortality in postmenopausal women, deaths, crude rate, and AAMR in the US, 1999-2023 AAMR: Age-adjusted mortality rate

State	Deaths	Crude Rate	AAMR	AAMR Lower 95% CI	AAMR Upper 95% CI	AAMR Standard Error
Alabama	2,303	15.22	15.12	14.5	15.74	0.32
Alaska	270	18.46	28.49	25.06	31.91	1.75
Arizona	2,329	12.2	12.24	11.75	12.74	0.25
Arkansas	1,170	12.71	12.06	11.36	12.75	0.35
California	24,250	24.37	23.19	22.89	23.48	0.15
Colorado	4,309	32.16	33.79	32.78	34.81	0.52
Connecticut	2,255	19.5	15.67	15.01	16.33	0.34
Delaware	397	13.51	13.53	12.19	14.86	0.68
District of Columbia	250	14.69	12.65	11.06	14.24	0.81
Florida	8,632	12.59	11.11	10.88	11.35	0.12
Georgia	2,999	11.76	12.84	12.38	13.3	0.24
Hawaii	1,611	37.86	31.92	30.34	33.5	0.81
Idaho	1,388	32.2	32.06	30.37	33.76	0.87
Illinois	7,854	20.96	18.46	18.05	18.88	0.21
Indiana	6,015	31.08	28.63	27.9	29.36	0.37
Iowa	3,289	33.35	25.63	24.73	26.53	0.46
Kansas	2,317	27.49	22.66	21.72	23.61	0.48
Kentucky	2,997	22.44	22.29	21.49	23.09	0.41
Louisiana	937	7.01	7.1	6.65	7.56	0.23
Maine	1,483	31.23	28.35	26.89	29.81	0.74
Maryland	4,308	25.1	24.36	23.62	25.09	0.37
Massachusetts	3,435	16.3	13.59	13.12	14.05	0.24
Michigan	5,932	19.01	17.5	17.05	17.95	0.23
Minnesota	8,482	54.52	45.42	44.43	46.41	0.5
Mississippi	1,079	12.17	11.92	11.2	12.63	0.36
Missouri	5,590	29.69	26.6	25.89	27.3	0.36
Montana	1,574	48.86	45.73	43.45	48.02	1.17
Nebraska	3,153	57.69	46.38	44.72	48.04	0.84
Nevada	652	8.9	10.94	10.09	11.78	0.43
New Hampshire	1,395	33.22	31.22	29.55	32.88	0.85
New Jersey	4,711	17.1	14.87	14.44	15.3	0.22
New Mexico	1,442	23.69	24.42	23.16	25.69	0.65
New York	6,409	10.52	9.15	8.92	9.37	0.12
North Carolina	6,882	23.88	24.06	23.49	24.63	0.29
North Dakota	929	44.42	32.76	30.58	34.94	1.11
Ohio	13,334	35.93	32.38	31.83	32.94	0.28
Oklahoma	2,923	25.86	24.71	23.81	25.61	0.46
Oregon	5,151	42.49	39.49	38.4	40.58	0.56
Pennsylvania	11,748	26.92	22.12	21.71	22.53	0.21
Rhode Island	933	26.66	20.16	18.83	21.49	0.68
South Carolina	2,865	19.44	20.5	19.75	21.25	0.38
South Dakota	1,097	43.34	33.15	31.13	35.17	1.03
Tennessee	4,996	25.4	25.38	24.67	26.08	0.36
Texas	14,304	22.94	24.1	23.7	24.49	0.2
Utah	1,768	30.98	32.42	30.91	33.94	0.77
Vermont	1,729	82.44	74.97	71.38	78.55	1.83
Virginia	4,533	19.53	19.69	19.12	20.27	0.29
Washington	6,578	33.93	32.71	31.91	33.51	0.41
West Virginia	1,893	28.94	27.61	26.36	28.86	0.64
Wisconsin	5,731	32.75	27.63	26.9	28.36	0.37
Wyoming	685	42.96	43.8	40.49	47.11	1.69
